# The Bristol Medical Library

**Published:** 1912-12

**Authors:** C. King Rudge

**Affiliations:** Honorary Librarian to the Bristol Medical Library


### THE BRISTOL MEDICAL LIBRARY.

BY

C. King Rudge, M.R.C.S. Eng., L.R.C.P. Lond.,
*Honorary Librarian to the Bristol Medical Library.*

---

In the *Journal* for December, 1911, there appeared an article from the pen of Mr. L. M. Griffiths on the history of the Library up to the year 1902. The present paper continues this subject, dealing with the growth and development of the Library during the past ten years.

This period has witnessed many important and critical changes in the constitution and general management of the Library, the principal of which are the outcome of a Report on the condition of the Library, drawn up by the Library Committee at the request of the Society and ratified by it in 1907. It was also accepted by the authorities of the University.

The preamble of the Report contains the following clause :—

> "The members of the sub-committee have endeavoured to make recommendations and suggestions, which, if carried out, will, in their opinion, make the Library more efficient and more worthy of so important a centre of medical education as Bristol ; but they have always borne in mind the original purposes of the Library ; and in proposing certain alterations which would necessitate considerable change in its constitution, they have never lost sight of the intention of the founders, and of the work of those whose untiring zeal and energy have built up the present excellent Library."

In accordance with the recommendations of the Report, a most important change was made in 1908, in the management of the Library. The Committee, while fully recognising the importance of a Reference Library, and after studying the conditions existing in twenty of the Medical Libraries in Great Britain which all allow their books to circulate among the members, decided that our members should have the privilege of borrowing books under certain restrictions without any increase in their annual subscription.

To meet the requirements of members who wish to see the most recent books as soon as they are published, the Society has become a subscriber to Lewis's Library.

That this endeavour to increase the efficiency and usefulness of the Library has in some degree been successful, is shown by the number of members who have availed themselves of the privileges by taking out books, and it is hoped that in the future there will be a large increase both in the number of borrowers and of the books borrowed.

The past year marks an eventful epoch in the annals of the Library, for after an occupation of nearly twenty years of the handsome and comfortable Reading Room and Library in the Medical Wing of the University, in the spring of last year notice was served on the Society by the University that their tenancy of the Medical Library would expire in March, 1912, as it was required for a Council Chamber, and at the same time the agreement between them would terminate. For various reasons the Society elected to forego the twelve months' notice, vacating the old Library in the early summer of 1911.

A joint committee was then appointed, composed of representatives of the University and Library Committee, to seek for suitable premises to serve for a library. After much consultation, the east wing of the Blind Asylum was fixed on for this purpose, as being the only available building large enough to accommodate the conjoint Library. This commodious building, after undergoing many necessary structural alterations and complete redecoration, to render it ready for the housing of our large and valuable collection of books and periodicals, was opened in October of last year for the use of members.

The Bristol Medical Library now occupies the east wing of the old Blind Asylum, which contains a suite of eight medium-sized and fairly-lighted rooms situated on two floors. On the ground floor is the Reading and Writing Room of the Medico-Chirurgical Society, a comfortable, well-lighted room ; on the walls of which are portraits of the past presidents of the Society and other eminent local members of the profession.

The entrance hall, now known as the "Current Periodical Reading Room," is well lighted and roomy. Here are placed the current numbers of the extensive series of journals and periodicals received by the Library.

The principal Reading Room and Library is on the floor above. It is a small, well-lighted room, the bookshelves of which are filled with the books most in requisition and recent accessions.

A special feature in the recent re-arrangement of books in the new Library may here be mentioned. In view of the increased accommodation, it has been possible to devote two rooms almost entirely to bound periodical literature. One room, containing most of the foreign journals and magazines, is located on the ground floor ; the other containing many complete sets of Transactions and other periodicals, mostly British, and is on the floor above. In this room are also found volumes in folio and quarto, and several valuable old medical works.

During the past ten years the growth and development of the Library in all departments has been well maintained, the number of volumes from all sources added during this period amounting to 5,947, the University contributing 2,010 volumes, and the Society 3,937. In estimating the growth of the conjoint libraries it is needful to take into consideration that 2,229 duplicate books have either been presented to other libraries or sold since 1902.

The members of the Society now have access to one of the largest and most up-to-date medical libraries out of London, containing over 22,000 volumes, with a list of 249 British and foreign periodicals.

At this stage of the history of the Medical Library, it is necessary to bear in mind what a most important part the *Journal* has played in building up and developing our valuable Library. It is not too much to say that the Library owes its very existence to the success of the *Journal*, and to those who in the past have raised it to such a high standard of literary efficiency. It now is the duty of the members of the Society to see to it that this standard is maintained, and that the circulation of the *Journal* is increased.

The *Journal* hands over to the Library of the Society all the books received for review. These, during the ten years from 1902 to 1912, reached a total of no less than 1,231 volumes, the value of which amounts to £346. The Library is also indebted to the *Journal* for the large number of Journals, 113, received in exchange. No further evidence is needed to demonstrate the substantial aid rendered to the Library by the *Journal*, and the active part it takes in its growth.

### THE FOLLOWING SETS OF PERIODICALS HAVE BEEN ADDED OR COMPLETED SINCE 1902.

*American Journal of Anatomy.*
*American Medicine.*
*Archives of the Middlesex Hospital.*
*Berliner klinische Wochenschrift.*
*Bulletin de la Société français de Dermatologie.*
*British Journal of Tuberculosis.*
*Canadian Medical Association Journal.*
*Dublin Hospital Reports.*
*Dublin Quarterly Journal of Medical Science.*
*Guy's Hospital Reports.*
*Journal of Experimental Physiology.*
*Journal of the American Medical Association.*
*Medical Review, The.*
*Philadelphia Medical Journal.*
*Progressive Medicine.*
*Proceedings of the Royal Society of Medicine.*

*Quarterly Journal of Medicine.*
*Reports of the Society for the Study of Children's Diseases.*
*Scottish Medical and Surgical Journal.*
*Transactions of the American Urological Society.*
*Virchow's Archiv.*
*West London Medical Journal.*

### THE FOLLOWING SETS ARE NEARLY COMPLETE.

*American Gynæcological and Obstetrical Journal,* Dec., 1891; June, 1892; Oct., 1897.

*American Journal of the Medical Sciences,* (N.S.), Nos. 1, 3–7, 1841–2.

*Annales de l'Inst. Pasteur,* Tomes i–vi.

*Archives of Pediatrics,* vols. i.–x., xii., No. 12.

*Asylum Journal of Mental Science,* Nos. 1–20, 1855–7.

*Brain,* pt. 32 (Jan., 1886).

*Dublin Journal of Medical Science,* July, 1880.

*Johns Hopkins Hospital Bulletin,* Vols. 1., ii. 1889–91.

*Johns Hopkins Hospital Reports,* Vol. i. 1889; vol. ii., Nos. 1–4, 1890.

*Journal of Anatomy and Physiology,* vols. 13, pts. 3, 4; 19, pts. 3, 4; 24, pt. 4; 26, pt. 1.

*Journal of Cutaneous and Venereal Diseases,* vols. i., ii. 1883–4.

*Journal of the Boston Society of Medical Sciences,* vols. i. ii.

*Journal of Laryngology,* vols. i.–iv.

*Journal of Mental Science.* Jan., 1861.

*Journal of Obstetrics and Gynæcology of British Empire,* vols. i., ii.

*Journal of Physiology,* vol. xxviii. pp. 181–257.

*Journal of the Royal Army Medical Corps,* vol. i.

*Journal of Tropical Medicine,* vol. i. No., 1.

*London Medical Review,* vol. i. 1860.

*Medical Directory,* 1862.

*Medical Facts and Observations,* vol. viii., 1800.

*Mitteilungen aus der medizinischen Fakultät der Kaiserlichen Universitat zu Tokyo,* bd. i.

*Provincial Medical and Surgical Journal,* vol. i., 1840–1.

*Revue de Laryngologie,* Tom. i.–x.

*Science Progress,* vol. i., 1906–07, Nos. 1, 2, 4.

*Surgery, Gynecology and Obstetrics,* vols. i. Nos. 1, 2, 3, 5, 6; ii. Nos. 1, 2, 4, 5, 6; iii.; v., No. 4.

*Trans. of the American Gynecological Society,* vols. i.–iii., ix.–xii.

*Trans. of the Association of American Physicians,* vols. i., iv.–viii.

*Trans. of College of Physicians of Philadelphia,* vol. x., 1888.

*Trans. of the Dermatological Society of Great Britain,* vol. viii.

*Trans. of the Edinburgh Obstetrical Society,* vols. iii., v., pt. 2.

*Trans. of the Medical Society of London,* vol. i., 1861.

*Zeitschrift fur Urologie,* bd. i., 1907.

### *TABLE SHOWING THE GROWTH AND DEVELOPMENT OF BRISTOL MEDICAL LIBRARY.*

1902 TO 1912.

| | | Oct.-Dec., 1902. | 1903. | 1904. | 1905. | 1906. | 1907. | 1908. | 1909. | 1910. | 1911. | 1912. |
|---|---|---|---|---|---|---|---|---|---|---|---|---|
| Books belonging to | Society .. .. .. | .. | 10087 | 10068 | 10453 | 10627 | 10915 | 11002 | 11098 | 11390 | 11619 | 11974 |
| ,, ,, ,, | University .. .. | .. | 7901 | 7852 | 7977 | 7804 | 7948 | 8445 | 8374 | 8477 | 8547 | 8645 |
| ,, ,, ,, | Bristol Royal Infirmary | .. | 2622 | 1822 | 1822 | 1800 | 1800 | 1192 | 1189 | 1189 | 1189 | 1189 |
| ,, ,, ,, | Bristol General Hospital . | .. | 1254 | 1022 | 1022 | 614 | 614 | 614 | 574 | 574 | 574 | 574 |
| Books sold or given to other Libraries by | University | 57 | — | 185 | — | 298 | 8 | 323 | 19 | — | — | 4 |
| | Society | 68 | 272 | 387 | 2 | 184 | 16 | 196 | 206 | — | 2 | 2 |
| Books received for Review .. .. .. | | .. | 126 | 119 | 97 | 118 | 126 | 101 | 108 | 130 | 75 | 120 |
| Journals regularly subscribed for by | University | .. | 27 | 27 | 27 | 29 | 29 | 32 | 32 | 34 | 34 | 34 |
| | Society | .. | 20 | 22 | 22 | 23 | 23 | 27 | 27 | 27 | 27 | 27 |
| Journals received by exchange or gift by | University | .. | 20 | 20 | 21 | 19 | 25 | 25 | 25 | 25 | 25 | 25 |
| | Society | .. | 169 | 167 | 156 | 160 | 160 | 160 | 165 | 163 | 163 | 163 |

BIBLIOGRAPHY OF THE BRISTOL MEDICAL LIBRARY.

*Felix Farley's Bristol Journal,* August 13th, 1831; July 2nd, 1836; July 1st, 1837.

*New Monthly Magazine,* September, 1831.

*Bristol Mirror,* July 1st, 1837.

*Bristol M.-Chir. J.,* 1890, viii. 287; 1891, ix. 66, 215, 246, 313; 1892, x. 284, 332; 1893, xi. 282; 1894, xii. 330; 1895, xiii. 316; 1896, xiv. 373; 1897, xv. 367; 1898, xvi. 369; 1899, xvii. 368; 1900, xviii. 102; 1901, xix. 284, 376; 1902, xx. 376; 1903, xxi. 379; 1904, xxii. 377; 1905, xxiii. 395; 1906, xxiv. 380; 1907, xxv. 382; 1908, xxvi. 369; 1909, xxvii. 379; 1910, xxviii. 376; 1911, xxix. 192, 335, 378.

